# Fussy

**Published:** 1873-01

**Authors:** 


### FUSSY.

They are perfectly exasperating,—are these fussy people; good natured, well meaning folks, to be sure, but then, they seem to be entirely deficient of all qualities which make the successful nurse so indispensable and ever welcome in the sick chamber.

You go home from your office with a "nervous headache,"—are tired and completely prostrated from the amount of brain-work there performed,—you want rest, and wish to be left entirely alone,—your ardent desire is not to be compelled to ask any questions, much less answer any. How confoundedly provoking is it then, to have every kind-hearted, but thoughtless, womanly member of your household, propound a lot of perplexing conundrums to you! "Won't you have a cup of tea?" "Sha'nt I get you a foot-bath?" "Let me rub your head,"—"Sha'nt I make you some milk toast?" "Would'nt you like a brandy sling?" "Shall I bring the big chair in for you?" "Would you like cold water on your forehead?" After a half-hour's bombardment of this character, in sheer desperation you cry, "no! no! no!" "I want to be let alone!" "Please close the door, keep the children quiet, darken the room and allow no one to approach." "I'm tired, have a nervous headache, and desire only quietness and rest."

If this is said in an impatient and emphatic manner—in keeping with one's state of mind, under this condition of affairs, you are left in perfect seclusion for a short interval, until some fussy person pokes her head through an open door way, just to see how you are getting along, or to fire another lot of questions at you. You dodge them as well as you can, relapse into another semi-conscious state, are about to fall to sleep, when another fussy appears upon the scene, feels of your feet, that they may not be cold, rubs your head, or performs some other little kindness that completely demoralizes you.

Perhaps both patient and attendant are fussy,—quite likely; but believe me, dear reader, the one with nervous headache is most to be pitied. If you have seen him once or twice in those terribly sick spells, you will have learned just what he requires, *without asking him a question.*

Remove his coat and boots. Place his wrapper on him; if his feet and hands are cold, place them in hot water for ten or fifteen minutes; then, wipe hands and feet perfectly dry, give him a cup of hot tea, darken the room, send the children out of hearing, and finally, stand guard outside the door, to prevent the approach of any one, and the better to hear his call when uttered. When that summons is delivered, you can then go in, to find him better; for depend upon it, a man with nervous headache, never opens his mouth to utter a syllable, until his distressing symptoms grow better. And this is the manner in which to treat such a customer; if you try it, we are sure you will be so pleased with its results, as never again to be fussy.

